# Regulatory T cell-deficient scurfy mice develop systemic autoimmune features resembling lupus-like disease

**DOI:** 10.1186/s13075-015-0538-0

**Published:** 2015-02-23

**Authors:** Eva N Hadaschik, Xiaoying Wei, Harald Leiss, Britta Heckmann, Birgit Niederreiter, Günter Steiner, Walter Ulrich, Alexander H Enk, Josef S Smolen, Georg H Stummvoll

**Affiliations:** Department of Dermatology, University of Heidelberg, Im Neuenheimer Feld 440, 69120 Heidelberg, Germany; Department of Pathology, Affiliated Zhong-Da Hospital, Southeast University, 87 Dingjia Bridge, Gulou, 210009 Nanjing, China; Department of Rheumatology, Medical University of Vienna, Wahringer Gurtel 18-20, 1090 Vienna, Austria; Department of Pathology, Hietzing Hospital, Wolkersbergenstrasse 1, 1130 Vienna, Austria

## Abstract

**Introduction:**

Scurfy mice are deficient in regulatory T cells (Tregs), develop a severe, generalized autoimmune disorder that can affect almost every organ and die at an early age. Some of these manifestations resemble those found in systemic lupus erythematosus (SLE). In addition, active SLE is associated with low Treg numbers and reduced Treg function, but direct evidence for a central role of Treg malfunction in the pathophysiology of lupus-like manifestations is still missing. In the present study, we characterize the multiorgan pathology, autoantibody profile and blood count abnormalities in scurfy mice and show their close resemblances to lupus-like disease.

**Methods:**

Scurfy mice have dysfunctional Tregs due to a genetic defect in the transcription factor Forkhead box protein 3 (Foxp3). We analyzed skin, joints, lung and kidneys of scurfy mice and wild-type (WT) controls by conventional histology and immunofluorescence (IF) performed hematological workups and tested for autoantibodies by IF, immunoblotting and enzyme-linked immunosorbent assay. We also analyzed the intestines, liver, spleen and heart, but did not analyze all organs known to be affected in scurfy mice (such as the testicle, the accessory reproductive structures, the pancreas or the eyes). We transferred CD4^+^ T cells of scurfy or WT mice into T cell-deficient B6/nude mice.

**Results:**

We confirm previous reports that scurfy mice spontaneously develop severe pneumonitis and hematological abnormalities similar to those in SLE. We show that scurfy mice (but not controls) exhibited additional features of SLE: severe interface dermatitis, arthritis, mesangioproliferative glomerulonephritis and high titers of anti-nuclear antibodies, anti-double-stranded DNA antibodies, anti-histone antibodies and anti-Smith antibodies. Transfer of scurfy CD4^+^ T cells (but not of WT cells) induced autoantibodies and inflammation of lung, skin and kidneys in T cell-deficient B6/nude mice.

**Conclusion:**

Our observations support the hypothesis that lupus-like autoimmune features develop in the absence of functional Tregs.

## Introduction

Scurfy mice have a missense mutation in the transcription factor Forkhead box protein 3 (Foxp3) gene and therefore lack functional CD4^+^Foxp3^+^ regulatory T cells (Tregs) and develop a lymphoproliferative disease with multiorgan inflammation, especially in the skin, the lung and the liver ([1,2]; reviewed in [3]). A main mediator of inflammation is the unrestrained activity of autoreactive CD4^+^ effector T (T_eff_) cells, which infiltrate tissues, recruit other inflammatory cells and ultimately lead to tissue damage [4]. Also, B cells are activated and high levels of immunoglobulins are present in the serum ([2,5]; reviewed in [3]). The contribution of B cells and autoantibodies for inflammation pathogenesis in scurfy mice was recently highlighted. B cell-deficient scurfy mice have less chronic inflammation and prolonged survival, and B cell transfer into these mice restores autoimmunity [6]. Interestingly, in the bone marrow, scurfy mice have fewer B cells and higher numbers of cells of the myeloid lineage as compared with wild-type (WT) littermates [7,8]. In a recent publication it was shown that these effects depend on granulopoietic effector cytokines (granulocyte macrophage colony-stimulating factor, tumor necrosis factor, interleukin 6 (IL-6)) and that Tregs do not directly affect B lymphopoiesis, but that they reduce the production of granulopoietic cytokines by suppressing the respective T_eff_ cells [7].

As their main effect, Treg cells are crucial for maintaining peripheral tolerance [9,10]. The most important subset are CD4^+^ cells that constitutively express the IL-2 α-chain (CD25) and Foxp3 [11]. Not only is Foxp3 a useful Treg marker (which allows differentiation from activated CD4^+^ T_eff_ cells), but its stable expression is required for Treg differentiation and function [12], as Foxp3 deficiency leads to a severe autoimmune-mediated multiorgan inflammation in mice [1,2] and to the related IPEX syndrome (immune dysregulation, polyendocrinopathy, enteropathy, X-linked) in humans [2,13-16]. Tregs mainly suppress T cells, but there is evidence that they also target a variety of other immune cells, such as B cells and dendritic cells (DCs) [9,11,17].

Scurfy mice develop a severe, generalized autoimmune disorder that can affect almost every organ system, including the conjunctiva, the liver and the reproductive system (testicles and accessory reproductive structures) [2,3,16]. Older reports also describe inflammation of the intestines and (as in human IPEX) of the pancreas, whereas newer ones do not [2,3]. Some of the autoimmune features in scurfy mice closely resemble those found in systemic lupus erythematosus (SLE), such as pneumonitis, whereas other typical characteristics of SLE, such as nephritis, have not been reported [2,3]. In line with this, impaired Treg function was observed in human SLE and decreased frequencies and function of Tregs correlate inversely with clinical disease activity ([9,18,19]; reviewed in [20]). In addition, a homeostatic imbalance of Tregs and conventional T cells has been described in experimental lupus, and the transfer of CD4^+^CD25^+^Foxp3^+^ Tregs was reported to prolong drug-induced remission [21,22].

As in scurfy mice, autoreactive T cells also play a central role in SLE pathogenesis *in vivo* because they are expanded, infiltrate affected organs and provide help for B cell activation. As a consequence, B cells are hyperreactive and produce (auto)antibodies [23-28].

In order to test the hypothesis that scurfy mice, as a consequence of their Treg deficiency, may exhibit a variety of autoimmune features resembling systemic lupus-like disease, we investigated these mice for typical manifestations of SLE. We analyzed for signs of nephritis, pneumonitis, arthritis and the occurrence of typical serum autoantibodies and reevaluated skin manifestations for lupus-like abnormalities. We also analyzed the intestines, liver, spleen and heart, but not all the other organs known to be affected in scurfy mice but not typically involved in SLE (such as the testicles or the accessory reproductive structures, the pancreas or the eyes) [16].

We show that Treg-deficient scurfy mice indeed share typical features of SLE, as they are positive for anti-nuclear antibodies (ANAs) and anti-double-stranded DNA (anti-dsDNA), anti-histone and anti-Smith (anti-Sm) antibodies (Abs). In addition, they are anemic and lymphopenic and develop pneumonitis, nephritis, arthritis and hyperkeratotic skin lesions that histologically resemble cutaneous lupus erythematosus. Furthermore, transfer of CD4^+^ T cells from scurfy mice, but not from WT controls, induced autoantibody production as well as pneumonitis, nephritis and severe skin disease in CD4^+^ T cell-deficient B6/nude mice.

Because scurfy mice exhibit more autoimmune features than are typical for SLE, we do not claim that the scurfy mouse is a lupus model. However, our experiments foster the hypothesis that lack of Treg function and the consequent lack of peripheral tolerance lead to systemic autoimmune features resembling those in SLE.

## Methods

### Scurfy mice

Female heterozygous B6.Cg-*Foxp3*^*sf*^/J (scurfy) mice were purchased from The Jackson Laboratory (Bar Harbor, ME, USA) and bred to C57BL/6 WT male mice to generate hemizygous male B6.Cg-*Foxp3*^*sf*^*/*Y (scurfy) offspring). C57BL/6 WT male littermates were used as controls.

As recipients for transfer experiments, B6.Cg-*Foxn1*^*nu*^/J (nude) (B6/nude) mice were purchased from The Jackson Laboratory. All mice were held under specific pathogen-free conditions at the central animal facility of the Interfacultary Biomedical Faculty, University of Heidelberg, Germany. Animal work was performed under the animal protocol (35-9185.81/G2010/10) approved by the local animal care committee (Regierungspräsidium Karlsruhe).

### Detection of autoantibodies

Serum samples were taken from scurfy and WT mice on day 21 of life. For evaluation of autoantibodies by immunofluorescence (IF), sera were diluted (as indicated in the figure legends) and added to slides precoated with either *Crithidia luciliae* (dsDNA) or HEp-20-10 cells and primate liver cells, respectively (ANAs) (all from EUROIMMUN, Lübeck, Germany). As a secondary Ab, goat-anti mouse immunoglobulin G (IgG) Alexa Fluor 488 (Invitrogen, Carlsbad, CA, USA), diluted 1:500 in phosphate-buffered saline (PBS), was used. For semiquantitative analyses, the slides were scored according to fluorescence intensity as follows: 0 = no positive staining, 1 = weakly positive staining, 2 = intermediate positive staining and 3 = strongly positive staining.

Anti-histone Abs were measured by enzyme-linked immunosorbent assay (ELISA) (Inova Diagnostics, San Diego, CA, USA). The results are presented in units per milliliter. Horseradish peroxidase–conjugated goat anti-rat Abs (1:2,000 dilution; SouthernBiotech, Birmingham, AL, USA) served as secondary Abs. Further analysis was performed by immunoblotting as described elsewhere [29,30].

### Hematological analysis

Scurfy and WT mice were bled at day 21 of life into tubes with sodium citrate to prevent clotting. Blood samples were immediately sent to the University of Heidelberg multidisciplinary center for blood analysis.

### Histological analysis of skin inflammation

When the mice were at day 21 of life, routine necropsies were performed for histopathologic evaluation, and skin tissue was fixed in 4% neutral buffered formalin. Fixed tissues were embedded in paraffin, and 5-μm sections were cut and stained with hematoxylin and eosin (H&E). Skin inflammation was scored in a graded fashion as previously described [31].

### Histological analysis of joints

Hind paws were prepared and analyzed by using previously described histopathologic techniques [32-35]. Staining with H&E allowed a general assessment, and toluidine blue (TB) destaining was performed to determine cartilage matrix loss. Tartrate-resistant acid phosphatase (TRAP) staining was performed to identify osteoclasts. Histomorphometric parameters (area of cartilage destruction, inflammation and erosion, as well as osteoclast numbers) were quantified by using the OsteoMeasure™ image analysis system (OsteoMetrics, Decatur, GA, USA).

Additional immunohistochemistry was done for T cells (anti-CD3; Novocastra Laboratories, Newcastle upon Tyne, UK), B cells (anti-CD45 receptor; BD Biosciences PharMingen, San Diego, CA, USA), macrophages (clone F4/80; AbD Serotec, Puchheim, Germany) and granulocytes (MCA771G; AbD Serotec) as reported previously [33-35], followed by quantitative analysis of the inflammatory infiltrate by tissue cytometry using HistoQuest™ software (TissueGnostics, Vienna, Austria) [36,37].

### Analysis of involvement of inner organs

Lungs, kidneys, spleens, hearts, intestines and livers were obtained from scurfy and WT mice; processed according to standard laboratory procedures; and stained with H&E. Kidney sections were also stained with periodic acid-Schiff (PAS) and analyzed by a blinded pathologist experienced in renal pathology of mice (WU).

For IF analysis, kidneys and back skin tissue samples were embedded in OCT compound and flash-frozen, and 5-μm cryosections were cut and fixed in acetone followed by 30 minutes of blocking with 5% goat serum in Tris-buffered saline (TBS). Slides were incubated with goat anti-mouse IgG Alexa Fluor 488 (Invitrogen) at 1:500 dilution in TBS for 1 hour in the dark.

Urinalysis was done by using the dipstick (Combur 5 Test HC; Roche Diagnostics, Mannheim, Germany) method with a semiquantitative system that allows scoring for erythrocyturia, leukocyturia and proteinuria from 0 (negative) to + (positive) and ++ (highly positive). For proteinuria, a score of at least ++ was considered pathologic because healthy mice also showed mild signs of proteinuria under these testing conditions (with a maximum of + positivity).

Scoring of pulmonary inflammation was based on a method published [38] and adapted [39] previously. In brief, for each vessel, we obtained a perimeter score according to the percentage of vessel perimeter surrounded by cells, calculated the mean width of the infiltrate (cell^F^ software; Olympus Soft Imaging Solutions, Münster, Germany) and multiplied these values for the final score [38].

### Transfer experiments

CD4^+^ T cells were isolated from lymph nodes of sick scurfy mice and male WT controls using magnetic activated cell sorting with CD4 microbeads (Miltenyi, Bergisch Gladbach, Germany). Purity of greater than 95% was confirmed by fluorescence-activated cell sorting analysis; CD4^+^ T cells were washed three times in PBS; and 2 × 10^6^ cells resuspended in 100 μl were injected into male B6/nude mice via tail vein injections. Four weeks after transfer, recipient mice were analyzed for autoantibody production (ANAs, anti-dsDNA Abs) for signs of inflammatory skin disease and for involvement of inner organs (lung and kidney) as described above.

### Statistical analysis

The data are expressed as mean ± SD. Student’s *t*-test or Fisher’s exact test (two tailed) was used for comparison of group values and discriminatory measures. One-way analysis of variance was used for repeated measurements of the same variable where appropriate. Wilcoxon’s matched-pairs test was used for the comparison of individual paired values if the distribution was not Gaussian. Significance was analyzed using Prism and InStat software (GraphPad Software, La Jolla, CA, USA), and *P*-values <0.05 were considered significant.

## Results

### Scurfy mice spontaneously develop a lupus-like skin phenotype

**Figure 1:**
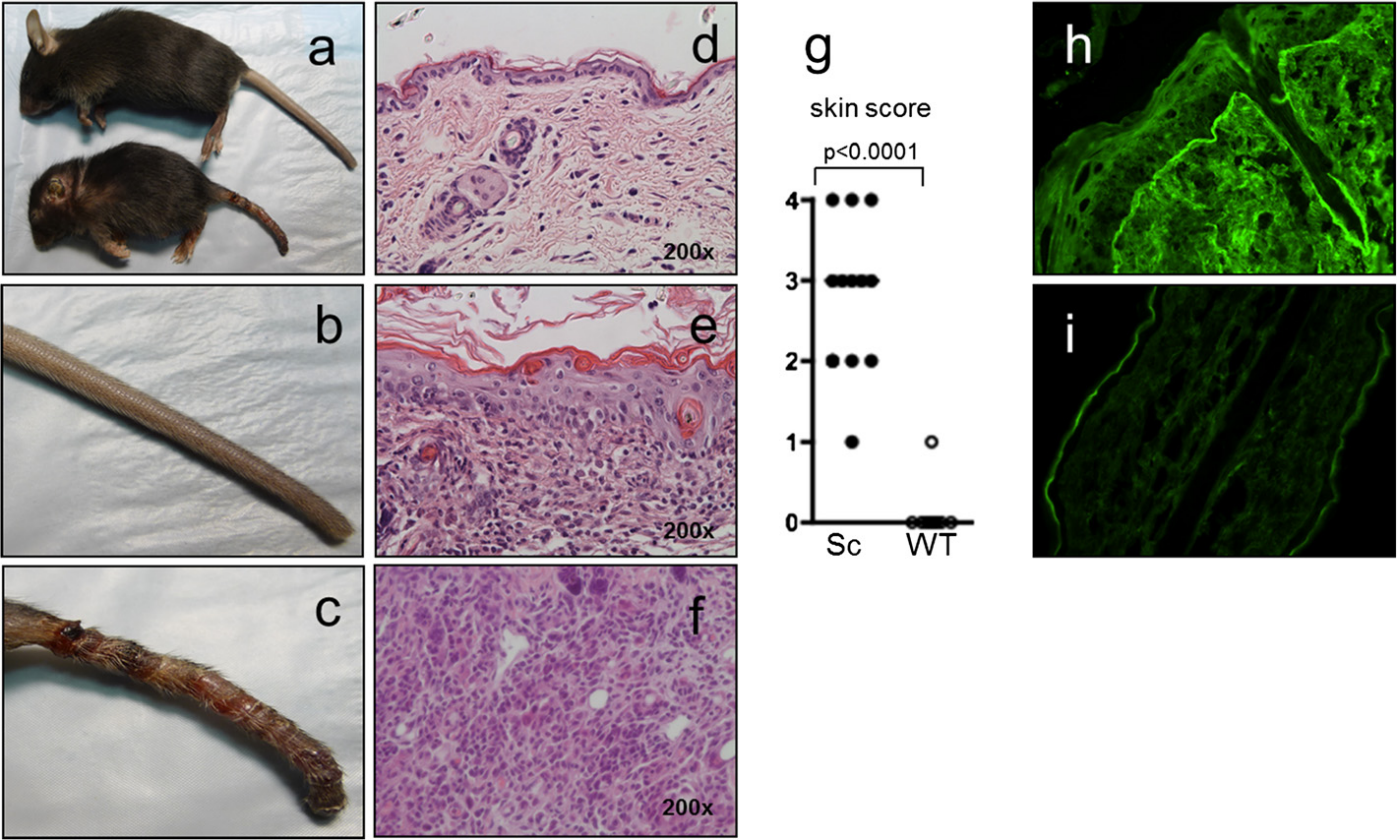
**Scurfy mice spontaneously develop severe autoimmune lupus-like skin inflammation.** We observed an inflammatory skin disease in scurfy mice that was a consequence of uncontrolled T cell expansion. A macroscopic view of scurfy (Sc) mouse and wild-type (WT) littermate control on day 21 of life **(a)** and a closer view of the tails of WT mouse **(b)** and scurfy mouse **(c)** are shown. Histological examination revealed interface dermatitis with effacement of the dermoepidermal junction and strong lymphohistiocytic inflammatory infiltrates, both of which are key histological features of cutaneous lupus erythematosus. Representative hematoxylin and eosin–stained sections of back skin of WT mouse **(d)** and sick scurfy mouse **(e)**, as well as a higher magnification image of inflammatory infiltrate in scurfy skin **(f)**, are also shown. **(g)** Summary of skin pathology scores of scurfy (*n* = 13) and WT (*n* = 13) back skin. Representative direct immunofluorescence images show scurfy **(h)** and WT **(i)** back skin tissue sections showing linear deposits of murine immunoglobulin only in scurfy skin.

Scurfy mice appear smaller than their WT littermate controls and have scaly skin on their ears, eyes and tails (reviewed in [3]). Around day 21 of life, scurfy mice are significantly smaller than normal and runted and exhibit severe skin inflammation (Figure 1a). In contrast to WT mice, the tail skin of scurfy mice is scaly and erythematous and reveals hyperkeratosis (Figure 1b,c). On histological analysis of inflamed scurfy back skin, the predominant features are interface dermatitis with effacement of the dermoepidermal junction (Figure 1e) and strong lymphohistiocytic inflammatory infiltrates (Figure 1f), both of which are histological features characteristic of cutaneous lupus erythematosus [40,41]. Using a previously described scoring system for skin pathology that comprises epidermal as well as dermal changes [31], we found significant skin inflammation in all scurfy skin samples, but no WT skin samples (Figure 1g). Using direct IF on cryosections of back skin, we found strong deposits of mouse IgG in the dermoepidermal basement membrane zone (similar to the lupus band observed in patients with SLE) in the skin of scurfy mice, but not in that of WT littermate controls (Figure 1h,i).

### Scurfy mice develop mesangioproliferative glomerulonephritis

**Figure 2:**
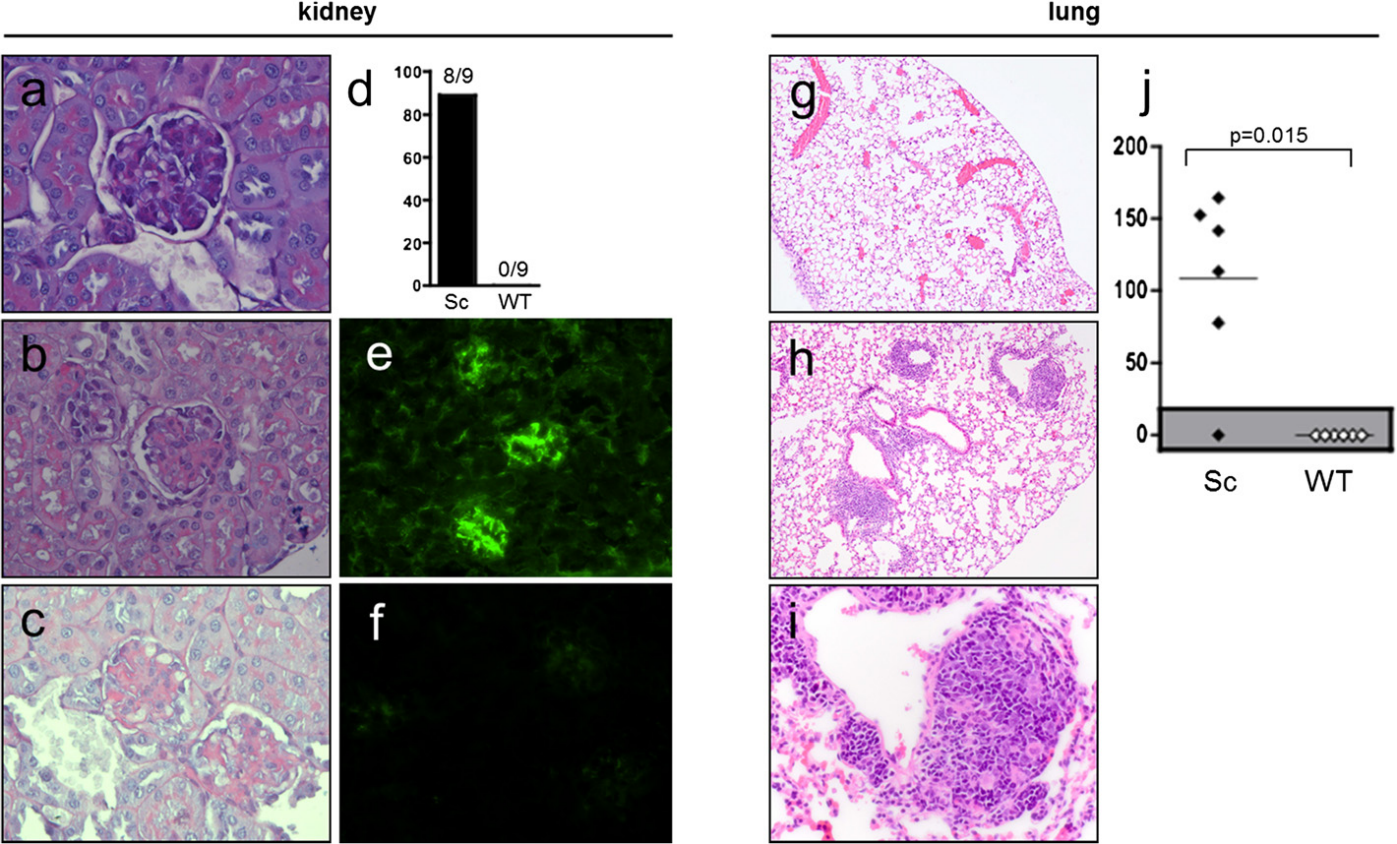
**Development of glomerulonephritis and pneumonitis in the majority of scurfy mice, but not in wild-type mice** ***.*** Scurfy (Sc) mice developed mesangial glomerulonephritis comparable to World Health Organization class II lupus nephritis in humans. Representative hematoxylin and eosin (H&E)–stained sections of a wild-type (WT) kidney **(a)** and a scurfy mouse kidney **(b)**, a periodic acid-Schiff stain of a glomerulum from a scurfy mouse kidney **(c)**. **(d)** Glomerulonephritis occurred in eight (89%) of nine scurfy mice, but not in controls (*P* = 0.0004). Lung involvement resembling lupus pneumonitis was found in all but one scurfy mouse (83%), but not in controls (*P* = 0.0152). Representative examples of direct immunofluorescence of a scurfy mouse kidney **(e)** and a WT kidney **(f)** are shown. Also shown are representative H&E-stained sections of a WT lung **(g)** and a scurfy lung **(h)**, a higher-magnification image of a peribronchial inflammatory infiltrate in a scurfy lung **(i)** and the total lung inflammation score in scurfy and WT mice **(j)**.

Kidneys sections were stained with H&E and PAS and analyzed by direct IF. All but one scurfy mouse developed mesangial glomerulonephritis (eight (89%) of nine) typical of World Health Organization (WHO) class II lupus nephritis, whereas the kidneys of WT mice were not affected (Figure 2a–d) [42]. On the basis of direct IF analysis, the majority of scurfy mice showed murine IgG deposits in the glomerula, whereas WT mice did not (Figure 2e,f). Urinalysis in a series of 14 scurfy mice showed proteinuria in 21% and erythrocyturia in 29% of mice, but no leukocyturia (data not shown).

### Pneumonitis in scurfy mice

All but one scurfy mice (five (83%) of 6), but no controls (zero of six) (*P* = 0.0152), developed pneumonitis characterized by alveolar wall thickening, interstitial edema and perivascular and peribronchial (lymphocyte enriched) inflammatory infiltrates and focal hemorrhages resembling human and murine lupus pneumonitis [39,43-45] (Figure 2g–j). In analyzing perivascular inflammatory infiltrates in scurfy lungs, we found that the mean perimeter score was 2.7 ± 1.5 and the width varied from 2 to 22 cell layers thick, with a mean of 47.4 ± 36.8 μm, leading to a significantly elevated total score (Figure 2j).

### Hyperreactive spleens and analysis of other inner organs

In line with the literature, we also found germinal center hyperplasia, depletion of lymphocytes in mantle zones and parafollicular areas of scurfy spleens, but not those of WT controls. In addition, scurfy mice, but not WT mice, showed periportal and perisinusoidal inflammatory infiltrates in the liver. There were no signs of inflammation in the intestines or the heart [1,2,16,31] (data not shown).

### Scurfy mice are anemic and lymphopenic

**Figure 3:**
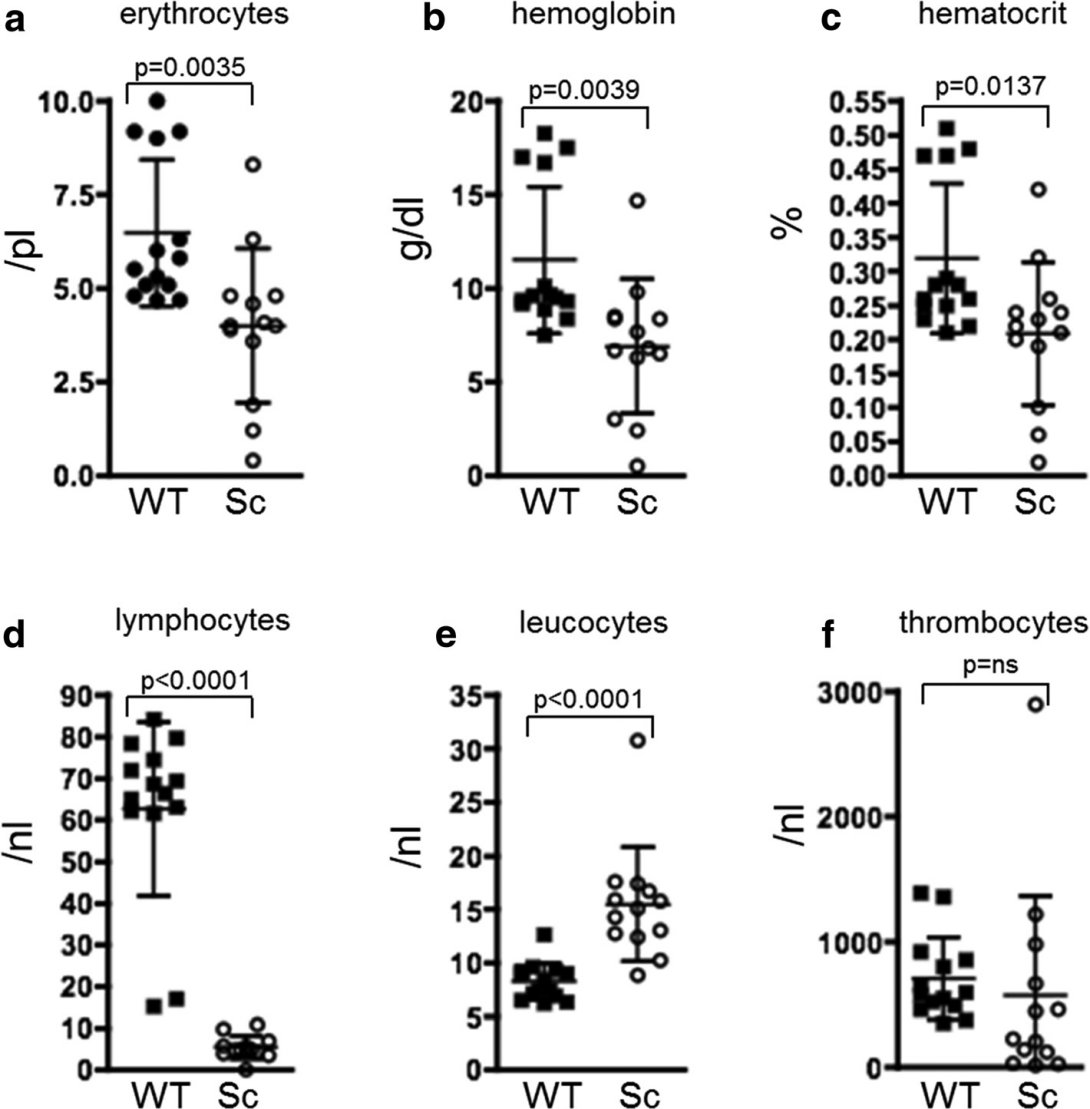
**Hematological analyses reveal anemia and lymphopenia in scurfy mice** ***.*** Freshly drawn blood of scurfy (Sc) and wild-type (WT) mice was immediately subjected to hematological analyses after being drawn. Scurfy mice were anemic, showing significantly reduced **(a)** erythrocytes, **(b)** hemoglobin and **(c)** hematocrit in comparison to WT controls. Upon analyzing the white blood cell count, we found elevated total leukocytes **(e)**, but reduced lymphocytes **(d)**, in scurfy blood, whereas thrombocytes did not show significant differences **(f)**. The results are shown as mean ± SD for scurfy mice (*n* = 13) and WT mice (*n* = 14) from two separate experiments. *P*-values are given in the figure.

Hemoglobin, hematocrit and erythrocyte counts were strongly reduced in scurfy blood in contrast to WT controls (Figure 3a–c). Complete blood cell counts revealed severe lymphopenia and leukocytosis in scurfy mice and slight (albeit not significant) changes in thrombocyte counts in comparison to WT mice (Figure 3d–f).

### Scurfy mice produce autoantibodies to nuclear antigens

**Figure 4:**
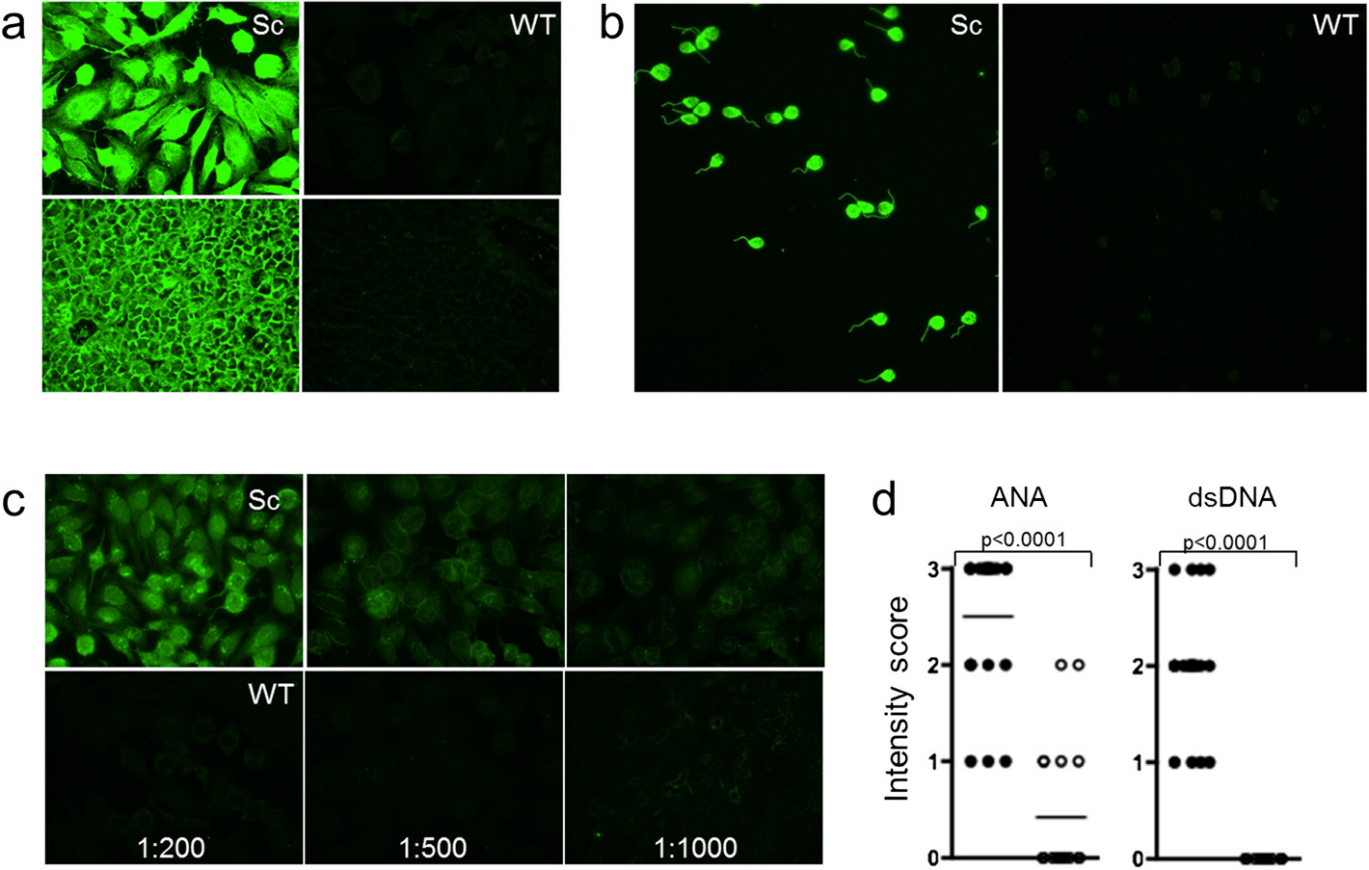
**High titers of antinuclear antibodies and anti-double-stranded DNA antibodies develop in the absence of functional regulatory T cells** ***.*** Antinuclear antibodies (ANAs) could be detected in all scurfy (Sc) sera when diluted 1:100 on slides coated with HEp-20-10 cells (upper panels) or primate liver tissue (lower panels) **(a)**. In addition, all scurfy, but no control (wild type (WT)), sera were positive for anti-double-stranded DNA (anti-dsDNA) antibodies (diluted 1:100) on slides precoated with *Crithidia luciliae* **(b)**. Examples of scurfy and WT sera at different dilutions **(c)** and a summary of the quantitative analysis of ANA and anti-dsDNA antibody positivity in scurfy and WT sera at the dilution of 1:100 **(d)** are given. The results are shown as mean ± SD for scurfy (*n* = 20) and WT (*n* = 20) mice. *P*-values are given in the figure.

All sera of scurfy mice contained not only ANA, but also anti-dsDNA-Abs. ANA staining showed a cytoplasmic and nuclear pattern and some samples were still positive at a dilution of 1:1000, while WT controls were negative for anti-dsDNA-Abs and had no or only weak staining for ANA (Figure 4a-c). Using a semiquantitative scoring system, scurfy showed significantly elevated values for both ANA and anti-dsDNA Abs when compared with controls (Figure 4d).

Scurfy mice also had significantly elevated serum levels of anti-histone Abs in ELISA analysis (145 ± 58 U/ml versus 66 ± 36 U/ml; *P* = 0.0031). A detailed immunoblot analysis of autoantibody suptypes revealed that 80% of scurfy (but not WT controls) had Abs against Sm antigen (8 of 10 versus 0 of 10; *P* = 0.0007) and that 90% were positive for U1 ribonucleoprotein (RNP) Abs (directed against RNP-A) (*P* = 0.0001), but did not develop Abs against Ro or La or against Scl-70 or Jo-1 antigens (data not shown).

### Scurfy mice show cartilage degradation and nonerosive arthritis

**Figure 5:**
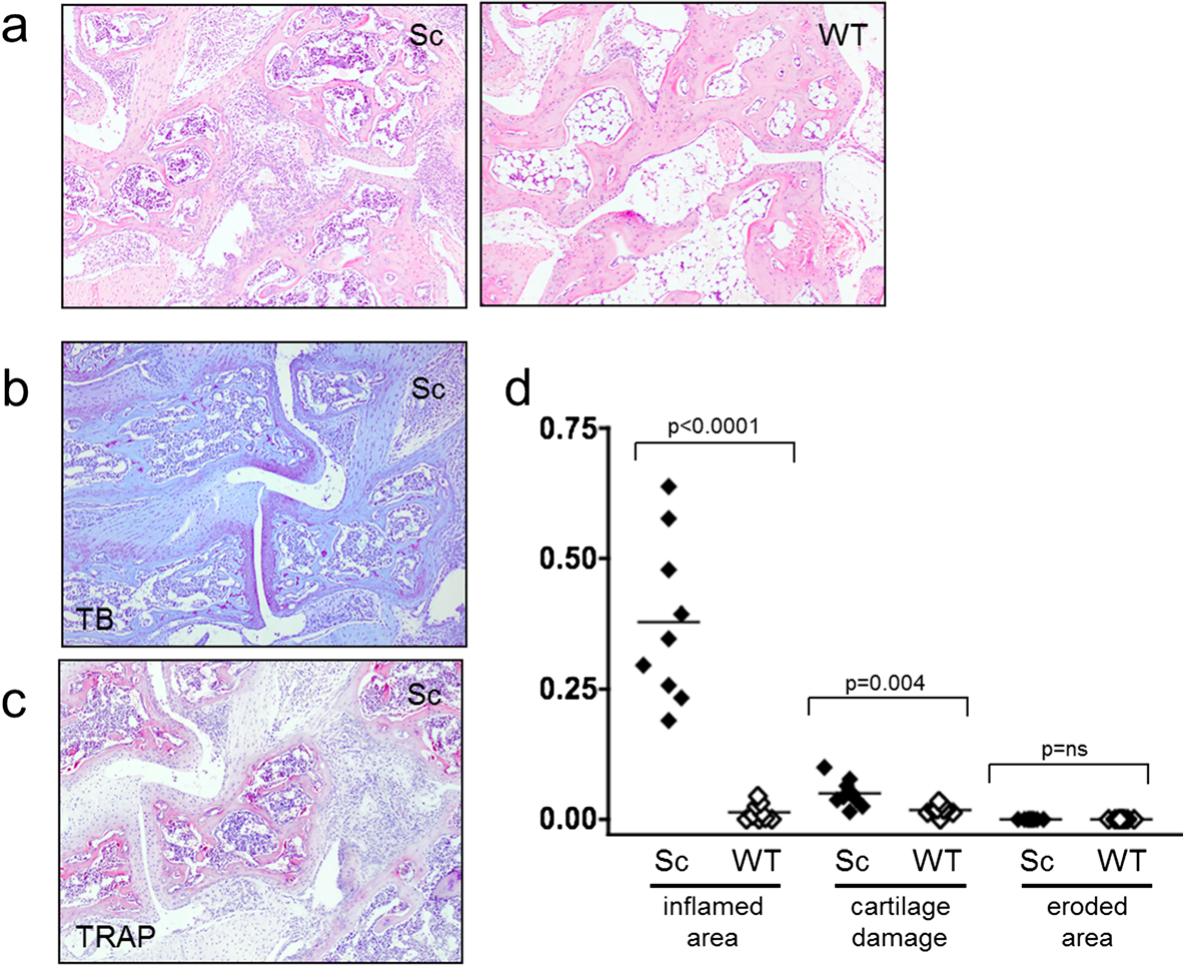
**Arthritis in scurfy, but not wild-type, joints** ***.*** All scurfy (Sc; nine of nine), but no control (wild type (WT); zero of nine) mice developed arthritis, as indicated by an inflammatory infiltrate (*P* < 0.0001). **(a)** Typical hematoxylin and eosin–stained cross-sections of the hind paws of a scurfy mouse (right panel) and a WT mouse (left panel) are shown. In addition, scurfy mice showed increased cartilage degradation (toluidine blue (TB) staining in **(b)**), but no osteoclasts were detected within the joint, and therefore no erosions were found (tartrate-resistant acid phosphatase (TRAP) staining in **(c)**). **(d)** Graphed data of the quantitative analysis of inflamed area, cartilage damage and eroded area in scurfy and WT mice are shown. The results are shown as mean ± SD for scurfy (*n* = 9) and WT mice (*n* = 9). *P*-values are given in the figure.

**Figure 6:**
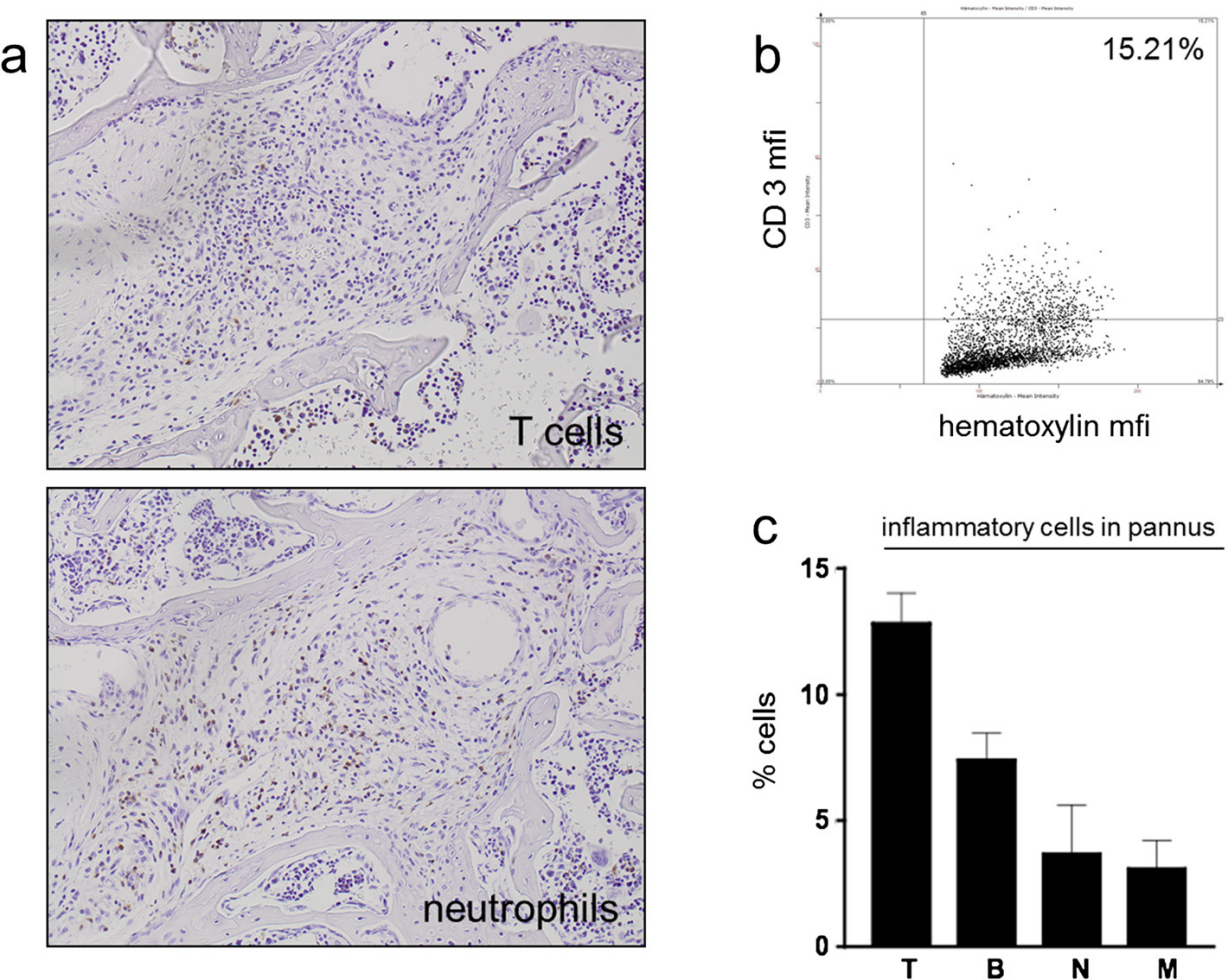
**T cells dominate the inflammatory infiltrate in arthritis in scurfy mice** ***.*** As a consequence of the unleashed T cell proliferation in this regulatory T cell (Treg)-deficient mouse model, the inflammatory infiltrate was dominated by T cells with lower frequencies of granulocytes. **(a)** A representative immunohistochemical analysis of the hind paw of a scurfy mouse stained with anti-CD3 (upper panel) and neutrophil marker anti-Ly6-B.2 (lower panel) is shown. The quantitative analysis of the inflammatory cellular infiltrate was done by tissue cytometry. A representative scurfy joint is shown in **(b)** and summarized in **(c)**. T, T cells; B, B cells; mfi, Mean fluorescence intensity; N, Neutrophils; M, Macrophages. The results are shown as mean ± SD for scurfy mice.

Clinically, the majority of scurfy mice showed moderate, diffuse swelling of the paws, which was hard to distinguish from subcutaneous edema. In histological analyses, scurfy mice showed increased cartilage degradation compared with WT controls, and they developed inflammatory infiltrates within the synovial membrane, whereas controls did not (Figure 5a,b,d). No osteoclasts were detected within the joint, and therefore no erosions were found (Figure 5c). Histomorphometric analysis revealed that (besides fibroblasts) the inflammatory infiltrate consisted mainly of CD3^+^ T lymphocytes (12.8 ± 2.1%) and B cells (7.4 ± 1.9), but also some neutrophils (3.7 ± 3.4%) and macrophages (3.1 ± 1.9) (Figure 6a–c).

### Transfer experiments

#### Scurfy CD4^+^ T cells induce autoantibody production in B6/nude mice

**Figure 7:**
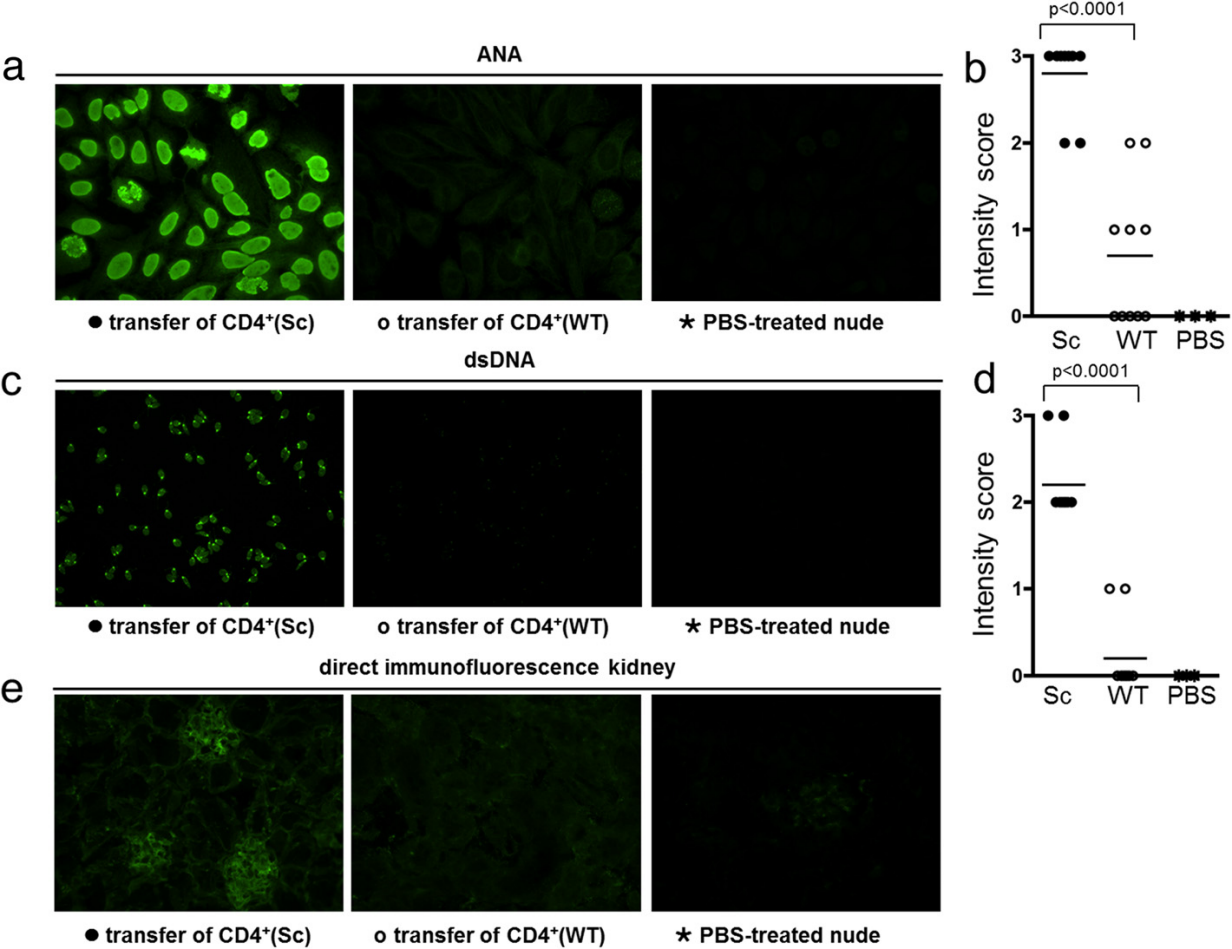
**Transfer of scurfy CD4** ^**+**^ **T cells induces autoantibody production in B6/nude mice.** Purified CD4^+^ T cells from lymph node single-cell suspensions of scurfy (Sc) and wild-type (WT) mice were transferred intravenously into immunocompromised B6/nude mice. Four weeks after transfer, the sera (diluted 1:100) of the recipient B6/nude mice were analyzed for the presence of autoantibodies. Antinuclear antibody (ANA) **(a and** **b)** and anti-double-stranded DNA (anti-dsDNA) antibodies **(c and** **d)** were positive in sera of B6/nude mice after transfer of scurfy CD4^+^ T cells, but could not be induced by transfer of WT CD4^+^ T cells or phosphate-buffered saline (PBS) injection. After transfer of scurfy CD4^+^ T cells from lymph node single-cell suspensions, kidneys of recipient mice showed murine immunoglobulin G deposits in glomerula, whereas recipient mice that received transferred WT CD4^+^ T cells or PBS did not **(e)**. **(a)**, **(c)** and **(e)** show one representative stain, and **(b)** and **(d)** summarize data of all mice from two independent experiments (*n* = 10 for scurfy or WT T cell-injected mice, *n* = 2 for PBS-injected mice). *P*-values are given in the figure.

To evaluate if autoreactive CD4^+^ T cells from scurfy mice could induce autoantibody production via T cell-mediated B cell help, we transferred purified CD4^+^ T cells from scurfy mice and WT controls into B6/nude mice, which completely lack CD4^+^ T cells but possess a normal B cell repertoire. Four weeks after transfer, we evaluated the presence of autoantibodies in the sera of recipient mice and found ANAs (ten of ten mice, 100%) and anti-dsDNA Abs (ten of ten, 100%) in B6/nude mice that received scurfy CD4^+^ T cells (Figure 7a,b). Among the B6/nude mice that received WT CD4^+^ T cells, only very few showed weak ANA positivity and only two mice showed very weak anti-dsDNA Abs (Figure 7c,d). These results indicate that, in the absence of functional Tregs, autoreactive CD4^+^ T cells expand and are able to induce ANAs as well as anti-dsDNA Abs via T cell-mediated B cell help.

#### Scurfy CD4^+^ T cells induce organ inflammation in B6/nude mice

**Figure 8:**
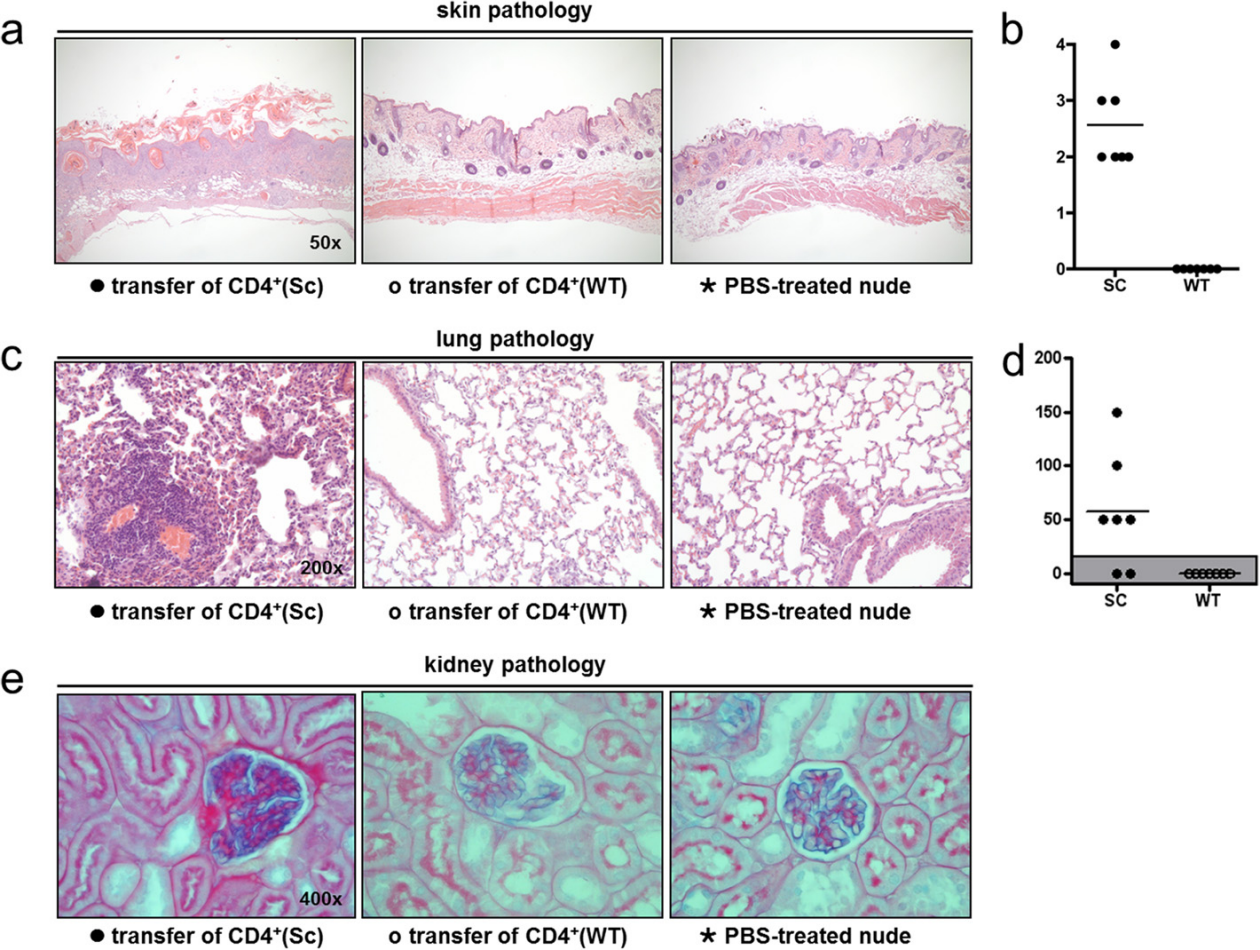
**Transfer of scurfy CD4** ^**+**^ **T cells induces skin, lung and kidney pathology in B6/nude mice** ***.*** Four weeks after transfer of purified CD4^+^ T cells from scurfy (Sc) and wild-type (WT) mice into immunocompromised B6/nude mice, tissues were analyzed by histology. All recipients of scurfy CD4^+^ T cells showed severe skin inflammation **(a and** **b)** and lung inflammation **(c and** **d)**, whereas recipients of WT CD4^+^ T cells and PBS-treated recipients did not. Kidney pathology was observed in two of seven recipients of scurfy CD4^+^ T cells, but in none of the controls **(e)**.

After transfer of CD4^+^ T cells from scurfy mice, but not of WT CD4^+^ cells or PBS, T cell-deficient B6/nude mice developed severe pneumonitis (seven of seven, 100%) and skin disease (seven of seven, 100%) resembling the disease spontaneously occurring in scurfy mice (Figure 8a–d).

In addition, four (57%) of seven mice that received scurfy CD4^+^ T cells developed proteinuria and erythrocyturia as observed by urinalysis after 4 weeks of CD4^+^ T cell transfer. Two of these mice also showed typical histological features of mesangioproliferative lupus nephritis in histological analysis (Figure 8e), whereas none of the control mice developed kidney disease.

## Discussion

Scurfy mice lack CD4^+^Foxp3^+^ Tregs and thus one crucial mechanism of peripheral tolerance. As a consequence, they die early as a result of a generalized systemic autoimmune disease [4]. Several autoimmune features of scurfy mice closely resemble those found in SLE, and Treg dysfunction has been reported in SLE [19]. Because the severe autoimmune disorder seen in scurfy mice (and in human IPEX) includes many more autoimmune features than are typical for SLE (including hypogonadism, pancreatitis, cholangitis and conjunctivitis), indicating overlap with other autoimmune diseases, the aim of the present study was not clearly at defining the scurfy mouse as a prototypical lupus model. Our aim was to evaluate the hypothesis that dysfunction of peripheral tolerance as a consequence of Treg malfunction (as seen in scurfy mice) leads to some of the typical features of SLE and therefore represents a lupus-like disease [9].

We confirm previous studies in showing that scurfy mice develop autoimmune characteristics compatible with SLE as pneumonitis, anemia, thrombocytopenia and inflammatory skin involvement [2,31]. –In addition, we report, as new findings underscoring our hypothesis, typical SLE-like phenomena such as glomerulonephritis, lymphopenia and nonerosive arthritis. Moreover, the intensive workup of scurfy skin showed that the cutaneous manifestations closely resemble those found in SLE, including the presence of linear IgG deposits resembling a lupus band. Most importantly, however, scurfy mice tested positive for the presence of ANA and anti-dsDNA autoantibodies (as well as for anti-histone- and anti-Sm Abs).

Pneumonitis is commonly observed in scurfy mice. Perivascular inflammation in scurfy lungs histologically resembles lung involvement in humans as well as that in murine lupus models [39,43-45]. As typically seen in SLE, scurfy mice developed mesangioproliferative glomerulonephritis meeting the criteria of WHO class II lupus nephritis, which may have been overlooked in previous analysis if sections were not specifically stained with PAS or by IF [42]. Scurfy mice also developed nonerosive arthritis characterized by a T cell-enriched synovitis and by cartilage damage, thus resembling the arthritic manifestations of human SLE [46]. Because of ethical issues related to severe lung involvement, scurfy mice must be analyzed within the first 3 to 4 weeks of life. Therefore, one can only speculate whether their arthritis would remain nonerosive or if osteoclasts would be attracted into the inflamed joint at a later time point [47].

In addition, we characterized the inflammatory skin disease as interface dermatitis with effacement of the dermoepidermal junction and strong lymphohistiocytic inflammatory infiltrates, both of which are key histological features of cutaneous lupus [40,41].

In line with our hypothesis, we found an SLE-typical autoantibody pattern with ANA and anti-dsDNA, anti-histone and anti-Sm Abs. These autoantibodies have escaped previous attention and could be detected in our study using sensitive techniques [2,48].

In transfer experiments, we could show that CD4^+^ T cells of scurfy mice, but not those of WT mice, transferred disease into T cell-deficient B6/nude recipients. This finding underscores the hypothesis that autoreactive CD4^+^ T cells expand in the absence of functional Tregs and that these cells are able to induce lupus-like pathology in lungs, skin and kidneys as well as production of typical autoantibodies via T cell-mediated B cell help. Interestingly, CD4^+^ T cells from scurfy lymph nodes can also induce myositis and inflammation of the salivary glands (resembling Sjögren’s syndrome) upon transfer into susceptible (RAG-1-knockout) recipients [49,50].

Thus, the systemic lupus-like features observed in scurfy mice appear to be a consequence of Treg dysfunction and uncontrolled CD4^+^ T_eff_ cell expansion, and, interestingly, a deficiency in Treg number and function has also repeatedly been postulated in human and murine SLE [21,51,52]. Therefore, the finding that scurfy mice present with many important characteristics of SLE supports the idea of a pathogenic role of Treg deficiency in this autoimmune disease. In line with this, the adoptive transfer of Tregs prevented the development of autoimmune disease in scurfy and had protective effects in lupus-prone mice [22,31,53,54].

As in SLE, there are signs of strong B cell activation in scurfy mice, as they have splenomegaly with germinal center hyperplasia and high levels of class-switched IgG autoantibodies [2]. In a recent publication, authors described using B cell-deficient scurfy mice to show that B cells are important for autoimmune pathology and that therapeutic B cell depletion decreased tissue pathology and increased survival [6]. The B cell activation observed in scurfy mice could be explained either by lack of direct suppression by Tregs or by increased helper stimuli provided by activated autoreactive CD4^+^ T cells. The latter view is supported by an observation that Treg-depleted *Foxp3*^*DTR*^ mice exhibit an expansion of follicular helper T cells that strongly augmented B cell proliferation, hyperimmunoglobulinemia and anti-dsDNA autoantibody production [55]. In line with these observations, our data show the induction of autoantibodies in recipient B6/nude mice after transfer of autoreactive scurfy CD4^+^ T cells, which proves that autoreactive CD4^+^ T cells expanding in the absence of functional Tregs are responsible for autoantibody production via T cell-mediated B cell help. These findings provide an important link from missing Treg control as present in scurfy mice to B cell-mediated autoimmune processes in SLE.

## Conclusions

The absence of functional Tregs is associated with the development of a multiorgan autoimmune disease due to activation of autoreactive CD4^+^ T cells and additional B cell-mediated disease. The developing autoimmune disease resembles human (and murine) SLE in many respects, although scurfy mice develop an even broader spectrum of autoimmune manifestations [3].

The data presented herein support the hypothesis that the lack of peripheral tolerance can lead to SLE-like features and thus underline an important role of Tregs in the pathogenesis of SLE, as suspected on the basis of previous reports on defective Treg function in active lupus [9]. The genetic defect in scurfy mice is precisely characterized and affects only Foxp3^+^ Tregs; consequently, all pathological features described in scurfy mice can be attributed to the lack of Treg control. Interestingly (as in SLE), we found not only direct T cell-mediated tissue inflammation but also B cell hyperreactivity and autoantibody production, which also fosters the idea that Tregs are important for maintaining peripheral tolerance against B cell-mediated autoimmunity.

## Abbreviations

Ab: Antibody
DC: Dendritic cell
ELISA: Enzyme-linked immunosorbent assay
Foxp3: Forkhead box protein 3
H&E: Hematoxylin and eosin
IF: Immunofluorescence
IgG: Immunoglobulin G
IL: Interleukin
IPEX syndrome: Immune dysregulation, polyendocrinopathy, enteropathy, X-linked
PAS: Periodic acid-Schiff
PBS: Phosphate-buffered saline
RNP: Ribonucleoprotein
SD: Standard deviation
SLE: Systemic lupus erythematosus
TB: Toluidine blue
TBS: Tris-buffered saline
T_eff_: Effector T cell
TRAP: Tartrate-resistant acid phosphatase
Treg: Regulatory T cell
WHO: World Health Organization
WT: Wild type
